# Synthesis and Catalytic Application of a Novel Recyclable Thermoregulated Phase-Transfer Phosphine Ligand

**DOI:** 10.3390/molecules31193454

**Published:** 2026-09-28

**Authors:** Yang Liu, Tianyuan Shao, Zhenghui Ma, Rui Wu, Guanghui Tian

**Affiliations:** 1 School of Chemistry and Environment Science, Shaanxi University of Technology, Hanzhong 723001, China; shaotianyuan@snut.edu.cn (T.S.); mazhenghui@snut.edu.cn (Z.M.); wurui@snut.edu.cn (R.W.); tiangh@snut.edu.cn (G.T.); 2 Shaanxi Key Laboratory of Catalysis, Shaanxi University of Technology, Hanzhong 723001, China

**Keywords:** thermoregulated phase-transfer, phosphine ligand, palladium catalyst

## Abstract

Organometallic complexes are widely used as efficient homogeneous catalysts in organic synthesis, but their recycling remains difficult. In this study, a novel L_TRPT22_-Pd(0) nanocatalyst with thermoregulated phase-transfer functionality was designed and synthesized, and its catalytic activity and recyclability in the Mizoroki–Heck reaction were investigated. The rationally designed phosphine ligand L_TRPT22_ contains a bulky electron-rich aryl moiety and flexible polyether chain segments, whose synergistic effect enables the resulting L_TRPT22_-Pd(0) nanocatalyst to combine the high activity of homogeneous catalysts with the facile recoverability of heterogeneous catalysts. The results showed that the L_TRPT22_-Pd(0) nanocatalyst exhibited excellent thermoregulated phase-transfer behavior in mixed aqueous/organic solvents, migrating to the organic phase upon heating and returning to the aqueous phase upon cooling, with a phase-transfer temperature of 47 °C. At 85 °C, the L_TRPT22_-Pd(0) nanocatalyst catalyzed the Mizoroki–Heck reaction of iodobenzene with butyl acrylate in a water/toluene biphasic system, affording a yield of over 92% (98% in pure toluene). The nanocatalyst could be recovered by simple phase separation and reused up to four times in this model reaction. These results demonstrate that the combination of steric and electronic modulation of the ligand with a thermoregulated phase-transfer functionalization strategy can overcome the intrinsic trade-off between high activity and easy recovery in conventional catalytic systems, thus providing a feasible approach for the development of novel efficient and recyclable palladium catalysts.

## 1. Introduction

The formation of carbon–carbon bonds through reactions catalyzed by Pd complexes has become a powerful tool in organic chemistry. Among these transformations, the Mizoroki–Heck coupling reaction has been widely applied in the synthesis of pharmaceuticals, agrochemicals, and functional materials, and is commonly used for the construction of substituted alkenes from aryl halides and alkenes [1,2]. The efficiency of this reaction largely depends on the nature of the ligands coordinated to the palladium center. In particular, phosphine ligands can significantly modulate the electronic properties, steric environment, and stability of the catalytically active Pd(0)/Pd(II) species [1,3]. Through rational ligand design, key steps in the catalytic cycle, such as oxidative addition, migratory insertion, and β-hydride elimination, can be markedly accelerated and/or better controlled in terms of selectivity. Compared with heterogeneous catalytic systems, homogeneous palladium–phosphine catalytic systems generally exhibit higher activity, but they suffer from the intrinsic drawback of difficult catalyst recovery [4,5]. Therefore, new ligand design strategies are required to overcome this limitation while maintaining high catalytic activity [6,7].

To address the long-standing trade-off between high activity and facile recovery of Pd catalysts, thermoregulated phase-transfer (TRPT) catalysis has been proposed as an attractive and versatile strategy [8,9]. The principle of TRPT catalysis is based on the temperature-dependent hydrogen-bonding interaction between the ligand and water molecules, which regulates the water solubility of the catalyst and endows it with reversible solubility between immiscible aqueous and organic phases [9,10]. Upon cooling, the catalyst is readily dissolved in the aqueous phase because of hydrogen bonding. Upon heating, disruption of these hydrogen bonds sharply decreases its solubility in water, causing the catalyst to transfer into the organic phase, where the catalytic reaction takes place. After completion of the reaction, cooling drives the catalyst back into the aqueous phase, thereby facilitating its separation and reuse. This reversible phase-transfer behavior enables TRPT catalysis to effectively combine the advantages of homogeneous and heterogeneous catalysis, while also allowing simple and rapid catalyst recovery [10]. Accordingly, the TRPT strategy has been successfully applied to a variety of catalytic transformations, including olefin hydrogenation, hydroformylation, and Pd-catalyzed cross-coupling reactions. In these reactions, the catalysts not only maintain good catalytic activity but also exhibit a certain degree of recyclability [9,11,12].

However, although TRPT catalysts have shown good activity and recyclability in various reactions, studies on the design and synthesis of TRPT phosphine ligands for Pd-catalyzed C–C coupling reactions remain rather limited [13]. The main reason is that the introduction of hydrophilic flexible chain segments to enhance the phase-transfer performance of the ligand may alter the electronic environment and steric structure around the metal center, thereby affecting the efficiency of key steps in the catalytic cycle, particularly oxidative addition and reductive elimination [13,14]. Therefore, achieving enhanced phase-transfer performance while maintaining effective control of catalytic activity by the phosphine ligand remains a key challenge in the design of TRPT phosphine ligands.

To address this issue, the phosphine ligand in this study was structurally designed from two perspectives, namely the regulation of catalytic activity and stability, and the introduction of TRPT properties. On the one hand, a bulky and electron-rich aryl structure was incorporated into the phosphine ligand. Electron-rich phosphine ligands can increase the electron density at the Pd center, thereby facilitating the oxidative addition of aryl halides. Large steric hindrance is also favorable for the formation of monodentate complexes and for the reductive elimination step, and can suppress cyclometalation and oxidation by O_2_ to some extent, thus improving catalyst stability [15,16,17]. On the other hand, flexible polyether chain segments were introduced into the ligand structure to endow the resulting metal nanocatalyst with TRPT behavior, thereby enabling efficient recovery after catalytic reactions in aqueous/organic biphasic systems [18].

Based on the above ligand design strategy, a TRPT phosphine ligand, L_TRPT22_ (Figure 1), was synthesized in this study. This ligand contains a bulky electron-rich arylphosphine framework and polyether chain segments. First, its TRPT behavior in a biphasic system was investigated. Next, a Pd catalyst was prepared through coordination of L_TRPT22_ with Na_2_PdCl_4_ followed by direct reduction without isolation of the intermediate. Finally, catalytic activity and recyclability were evaluated using the Mizoroki–Heck coupling of iodobenzene with butyl acrylate as a model reaction. By combining steric/electronic tuning of the ligand with the introduction of polyether chain segments bearing phase-transfer functionality, both high catalytic activity and facile recoverability of the palladium catalyst were achieved. This study therefore provides a feasible approach to the recycling of Pd catalysts and expands the library of functional phosphine ligands.

## 2. Results and Discussion

### 2.1. Visual Phase-Transfer Process of the L_TRPT22_-Pd(0) Nanocatalyst

At room temperature, it could be observed that after partitioning in a water/toluene biphasic solvent system, the nanocatalyst formed from the reaction of L_TRPT22_ with Na_2_PdCl_4_ exhibited the characteristic color of the L_TRPT22_-Pd(0) nanocatalyst only in the aqueous phase, whereas the toluene phase was nearly colorless (Figure 2a). When the temperature of the biphasic system was raised above 47 °C, a clear change in the distribution of the L_TRPT22_-Pd(0) nanocatalyst between the two phases was observed. The color of the aqueous phase gradually faded and finally disappeared, while the color of the organic phase gradually deepened until it became the characteristic color of the palladium nanocatalyst, indicating that the L_TRPT22_-Pd(0) nanocatalyst had migrated from the aqueous phase to the organic phase (Figure 2b). When the system was cooled back to room temperature, the L_TRPT22_-Pd(0) nanocatalyst returned to the aqueous phase, and the initial phase distribution was restored (Figure 2c). In addition, the L_TRPT22_-Pd(0) nanocatalyst exhibited the same thermoregulated phase-transfer behavior in a *n*-pentanol/water biphasic system. At room temperature, the nanocatalyst was distributed in the aqueous phase, whereas when the temperature was increased above 47 °C, it transferred to the organic phase and returned to the aqueous phase again upon cooling. This temperature-dependent phase-migration process could be repeatedly achieved by heating and cooling, indicating that the L_TRPT22_-Pd(0) nanocatalyst possessed reversible thermoregulated phase-transfer properties.

In the above thermoregulated phase-transfer process, the reason why the L_TRPT22_-Pd(0) nanocatalyst was located in the lower aqueous phase at room temperature is that the polyether chain in the phosphine ligand contains numerous oxygen atoms, which can form extensive hydrogen-bonding interactions with water molecules at room temperature, thereby imparting hydrophilicity and allowing the L_TRPT22_-Pd(0) nanocatalyst to dissolve well in the aqueous phase. When heated above 47 °C, these hydrogen-bonding interactions were disrupted, and the L_TRPT22_-Pd(0) nanocatalyst became lipophilic and transferred to the upper organic phase. Upon cooling back to room temperature, the nanocatalyst returned to the lower aqueous phase owing to the re-formation of hydrogen bonds with water molecules. This thermoregulated phase-transfer process constitutes the basis for the use and recovery of the L_TRPT22_-Pd(0) nanocatalyst as a catalyst in biphasic solvent systems.

### 2.2. Effect of Solvents on the Catalytic Activity of the L_TRPT22_-Pd(0) Nanocatalyst

Compared with conventional Homogeneous catalytic reactions conducted in a single organic solvent, the use of the L_TRPT22_-Pd(0) nanocatalyst as a catalyst requires a biphasic solvent system to facilitate catalyst recovery, and this change may have a significant influence on catalytic activity. Therefore, the effect of the solvent system on the catalytic activity of the L_TRPT22_-Pd(0) nanocatalyst was investigated using the Mizoroki–Heck coupling of iodobenzene with butyl acrylate as a model reaction, and the results are shown in Figure 3.

The results showed that toluene was more suitable as the organic phase of the solvent system. When toluene was used as the sole solvent, the coupling reaction afforded the target product in a high yield (98.3%). However, after completion of the reaction, the catalyst tended to aggregate because of its poor solubility in toluene. In addition, recovery of the catalyst by adding water after the reaction was cumbersome and time-consuming, and could easily lead to catalyst deactivation and loss. It was found that when water was introduced at the beginning of the reaction, the catalyst became uniformly distributed in the aqueous phase after the reaction and was less prone to aggregation. Therefore, an organic/water biphasic system was employed as the reaction medium in the subsequent studies. When *n*-pentanol was used as the sole solvent, the reaction efficiency decreased markedly. Moreover, the reaction efficiencies in the *n*-pentanol/water biphasic solvent system and in pure water were both lower than that in pure *n*-pentanol. These changes in reaction efficiency were mainly related to the solubility of both the catalyst and the reactants in the reaction medium. During the reaction, low-polarity toluene provided better solubility for the catalyst and reactants, which was beneficial to the progress of the reaction. In contrast, highly polar solvents or water-rich environments were unfavorable for the dissolution and dispersion of the catalyst and reactants, resulting in decreased catalytic activity and reaction efficiency. On the basis of these results and considering catalyst recovery, the toluene/water biphasic system was selected as the solvent for subsequent catalytic and recycling studies.

### 2.3. The Influence of CuI on Catalytic Activity

It should be noted that CuI was introduced as a co-catalyst based on previous reports, which indicated that the presence of CuI could slightly improve the yield and lead to a cleaner reaction for the Heck coupling of iodonucleoside analogues [19,20]. To verify the role of CuI in the catalytic system, a control experiment without the addition of CuI was performed under identical conditions. The results of the first run showed that while the coupling reaction could still proceed in the absence of CuI (yield 52%), the yield of the target product decreased significantly compared to the reaction with CuI (yield 91%). This result confirms that CuI acts as a co-catalyst in the present system, effectively improving the reaction efficiency.

### 2.4. Recycling of the L_TRPT22_-Pd(0) Nanocatalyst Catalyst

Based on the thermoregulated phase-transfer behavior and catalytic activity of the L_TRPT22_-Pd(0) nanocatalyst, its recyclability was further evaluated using the Mizoroki–Heck coupling of iodobenzene with butyl acrylate as a model reaction. After each run, the reaction mixture was cooled to 20 °C, whereupon the L_TRPT22_-Pd(0) nanocatalyst returned to the aqueous phase. It was found that, after decantation of the organic phase, the remaining aqueous phase could be directly reused in the next run without any further treatment.

As shown in Figure 4, the catalyst still exhibited good catalytic activity after multiple recycling runs. The yield in the initial run was 91%, and the catalyst showed comparable performance in the subsequent three runs, giving yields of 89%, 85.6%, and 80.9%, respectively. The corresponding TOF values over the four runs were 34.2, 33.4, 32.1, and 30.4 h^−1^, respectively, showing a gradual downward trend. This decline was highly consistent with the change in product yield, indicating that the catalyst underwent slow deactivation during repeated use. It is worth noting that the TOF values were calculated based on the actual molar amount of the catalytically active L_TRPT22_-Pd(0) nanocatalyst formed, which is determined by the amount of L_TRPT22_, rather than the total amount of Pd(0). In the TRPT catalytic system, only the Pd(0) coordinated with the L_TRPT22_ ligand can migrate into the organic phase to catalyze the reaction. Uncoordinated Pd(0) tends to aggregate into metallic clusters and remains in the aqueous phase, unable to exert catalytic activity. Therefore, calculating the TOF values based on the actual amount of L_TRPT22_ provides a more accurate reflection of the catalytic efficiency of this catalyst system. Although there is still room for further reduction in the Pd loading in our system, which is 4 mol% compared to the 10 mol% Pd used in the scaled-up Heck alkylation reaction reported by Bhilare et al. [21], its main advantage lies in the ease of catalyst separation and recovery through temperature-controlled phase-transfer technology, rather than maximizing the activity per single run.

### 2.5. UV–Vis Absorption Spectral Analysis

Using L_TRPT22_ as the ligand and Na_2_PdCl_4_ as the palladium source, the L_TRPT22_-Pd(0) nanocatalyst was prepared by H_2_ reduction. Figure 5 shows the UV–Vis absorption spectra of Na_2_PdCl_4_ and L_TRPT22_ in H_2_O before and after the reduction reaction. Before reduction, the absorption peak at 365 nm arose from charge transfer between the ligand L_TRPT22_ and Pd(II), indicating the formation of the L_TRPT22_-Pd(II) complex. Specifically, this near-UV absorption band can be reasonably assigned to the ligand-to-metal charge transfer (LMCT), which is consistent with the spectral behavior of square-planar Pd(II)-phosphine complexes reported in the literature [22,23,24]. After reduction, this characteristic absorption peak disappeared, indicating that Pd(II) had been directly converted into Pd(0), leading to the formation of the L_TRPT22_-Pd(0) nanocatalyst.

### 2.6. ICP-OES Analysis of Pd Leaching After Recycling

To evaluate the metal leaching of the L_TRPT22_-Pd(0) catalyst during the recycling process, the Pd content in the upper toluene phase was determined by ICP-OES after five consecutive Mizoroki–Heck reactions. Specifically, the same batch of catalyst was continuously used for five runs; after the fifth run, the system was cooled to room temperature and allowed to separate, and the upper toluene phase was then collected for ICP-OES analysis. The results showed that the Pd content detected in the upper toluene phase was 5% of the initial Pd loading. This result indicates that a certain degree of Pd leaching occurred during the catalyst recycling, which could be one of the reasons for the decline in yield during the recycling runs [25].

### 2.7. Changes in Particle Size and Dispersity of the L_TRPT22_-Pd(0) Nanocatalyst Before and After Recycling

To characterize the changes in particle size and dispersity of the L_TRPT22_-Pd(0) nanocatalyst before and after use, its micromorphology was investigated using transmission electron microscopy (TEM). As shown in Figure 6a for the freshly prepared catalyst, the Pd nanoparticles (NPs) obtained after high-pressure H_2_ reduction are predominantly near-spherical. They are uniformly dispersed within the observed field of view, with no obvious continuous film formation or bulk agglomeration. The corresponding particle size distribution histogram on the right shows a relatively narrow size distribution, with a statistically determined average diameter of approximately 1.7 nm. This indicates that the obtained Pd NPs possess a small overall size and good dispersity. This result can likely be attributed to the steric hindrance provided by the isopropyl substituents on the biphenyl backbone, combined with the stabilizing effect of the PEG chains; their synergistic effect helps inhibit the aggregation of Pd NPs during the reduction process. The small particle size and good dispersion state also facilitate the exposure of active sites, laying the foundation for its subsequent application in cross-coupling reactions. The characterization results demonstrate that L_TRPT22_ exerts a robust stabilizing effect on the Pd NPs, enabling the catalyst to exist with a small particle size and uniform distribution, which provides favorable conditions for evaluating its subsequent thermoregulated phase-transfer behavior and catalytic performance.

To further evaluate the structural stability of the L_TRPT22_-Pd(0) nanocatalyst during recycling, TEM characterization was performed on the catalyst after four consecutive cycles (Figure 6b). As illustrated, the recycled Pd NPs remain relatively uniformly dispersed in the system. No significant changes in overall morphology or large-scale severe agglomeration were observed. Statistical analysis reveals that the average diameter of the Pd NPs after four cycles is 2.2 ± 0.3 nm. Compared to the fresh catalyst, the particle size of the recycled Pd NPs increased only slightly. This indicates that the L_TRPT22_ ligand maintains a strong stabilizing effect on the Pd NPs during the repeated heating-cooling and phase-separation recovery processes, effectively suppressing metal particle aggregation. Combined with the catalytic recycling results, the catalyst retains good catalytic activity after multiple reuses. The slight decrease in activity may be attributed to the minor increase in particle size and the slight decrease in dispersity of a fraction of the Pd NPs during the cycling process.

## 3. Experimental Section

### 3.1. Reagents and Instruments

Na_2_PdCl_4_ (≥98%) and polyethylene glycol monomethyl ether (PEG-OMe, Mn = 1000) were purchased from Shandong Keyuan Biochemical Co., Ltd., Laizhou, China; iodobenzene (≥99%) and triethylamine (≥99%) were purchased from Sinopharm Chemical Reagent Co., Ltd. Shanghai, China; butyl acrylate (≥99%) was purchased from Shanghai Macklin Biochemical Co., Ltd., Shanghai, China; copper(I) iodide (CuI, ≥98%) was purchased from Shanghai Aladdin Biochemical Technology Co., Ltd., Shanghai, China; H_2_ and Ar were supplied by Hanzhong Xinghui Gas Station. All reagents were used as received without further purification. Tetrahydrofuran was refluxed over sodium and distilled prior to use. *n*-Hexane, dichloromethane, toluene, diethyl ether, and other organic solvents were distilled and purified before use. The water used in the experiments was ultrapure water produced by a UP ultrapure water system.

SCR1 thermostatic magnetic stirrer (BIOCOTE Ltd., Coventry, UK); ADVANCE 600 fourier digital NMR spectrometer (BRUKER, Fällanden, Switzerland); VERTEX70 infrared spectrometer (BRUKER, Fällanden, Switzerland); UV-2600 UV–Vis spectrophotometer (Shimadzu, Japan); DFY-5/80 low-temperature thermostatic reaction bath (Gongyi Yuhua Instrument Co., Ltd., Gongyi, China); 7890B-5977B gas chromatography–mass spectrometry system (Agilent, Santa Clara, CA, USA); JEM-2100F transmission electron microscope (JEOL Ltd., Akishima, Tokyo, Japan).

### 3.2. Experimental Procedure

#### 3.2.1. Synthesis and Characterization of PEG-SE and L_TRPT22_

##### Synthesis of PEG-SE

The synthetic route of PEG-SE is shown in Scheme 1.

Polyethylene glycol monomethyl ether (PEG-OMe, 12.50 g, 12.48 mmol) was added to a reaction flask that had undergone three to five vacuum/argon refill cycles and was dissolved in dehydrated and deoxygenated toluene (20 mL). Triethylamine (1.52 g, 1.2 equiv) was then added. Ethanesulfonyl chloride (1.61 g, 1.0 equiv) was dissolved in anhydrous toluene (5 mL) and transferred into a pressure-equalizing dropping funnel connected to the reaction flask. Under ice-bath cooling and magnetic stirring, the ethanesulfonyl chloride solution was slowly added dropwise to the PEG-OMe solution over about 10 min. After the addition was complete, the reaction mixture was stirred in the ice bath for 3 h and then allowed to warm naturally to room temperature and stirred overnight. After completion of the reaction, deionized water (250 mL) was added to dilute the system, and the mixture was extracted with dichloromethane (50 mL × 5). The combined organic phases were washed successively with 1 M HCl (50 mL × 4) and saturated brine (50 mL × 4). The organic layer was dried over anhydrous Na_2_SO_4_ and concentrated under reduced pressure to remove most of the solvent. An appropriate amount of diethyl ether was then added to the concentrated mixture, and the product was precipitated by sonication. The resulting solid was collected by filtration, washed with an appropriate amount of diethyl ether, and finally dried under vacuum at room temperature for 6 h to afford PEG-SE as a white solid (13.38 g, 98% yield) [26].

##### Synthesis of L_TRPT22_

The synthetic route of L_TRPT22_ is shown in Scheme 2.

##### Synthesis of Intermediate 1

Under an N_2_ atmosphere, 2-bromo-2′,4′,6′-triisopropyl-3,6-dimethoxy-1,1′-biphenyl (5.00 g, 12.55 mmol) was dissolved in anhydrous tetrahydrofuran (THF, 20 mL), and the solution was cooled to −78 °C. A solution of *n*-butyllithium in *n*-hexane (1.6 M, 7.84 mL, 12.55 mmol) was then added dropwise. After the addition was complete, the reaction mixture was stirred at −78 °C for a further 1.5 h to generate the corresponding aryllithium intermediate 1. This intermediate was used directly in the next step without isolation [27].

##### Synthesis of Intermediate 2

In a dry 100 mL three-necked flask equipped with a magnetic stir bar and purged with argon, phenyldichlorophosphine (1.62 mL, 11.95 mmol) was dissolved in anhydrous THF (10 mL), and the solution was cooled to −78 °C for 15 min. Subsequently, the above-prepared THF solution (27.84 mL) of the aryllithium reagent (11.92 mmol) was slowly added dropwise to the phenyldichlorophosphine solution at −78 °C through a double-ended needle, with the addition rate carefully controlled. After the addition was complete, the reaction mixture was maintained at −78 °C for 2 h, then allowed to warm slowly to room temperature and stirred overnight to afford intermediate 2 [28].

##### Synthesis of Intermediate 3

Under an Ar atmosphere, a suspension of LiAlH_4_ (0.455 g, 11.92 mmol) in anhydrous THF (4.76 mL, 2.5 M) was cooled to 0 °C in an ice bath. The THF solution (30 mL) of intermediate 2 prepared in the previous step was then added dropwise to the above suspension with stirring. After the addition was complete, the reaction mixture was stirred at 0 °C for 30 min. The ice bath was then removed, and the reaction mixture was allowed to warm naturally to room temperature and stirred overnight. After completion of the reaction, the volatile components were removed under reduced pressure. A mixed solvent of *n*-hexane and anhydrous diethyl ether was added to the residue, and the insoluble aluminum salts were removed by filtration through a fritted glass funnel under strictly air-free conditions. The resulting filtrate was concentrated under reduced pressure to afford crude intermediate 3 (5.2 g), which was used directly in the next step without further purification [29].

##### Synthesis of the Target Molecule L_TRPT22_

Under an Ar atmosphere, intermediate 3 (5.5 g, 11.92 mmol) was dissolved in anhydrous THF (40 mL) in a dry 250 mL three-necked flask and cooled to −78 °C. A solution of *n*-butyllithium in *n*-hexane (1.6 M, 7.45 mL, 11.92 mmol, 1.0 equiv) was then added dropwise. After the addition was complete, the reaction mixture was stirred at −78 °C for a further 1.5 h to generate the corresponding lithium phosphide solution. PEG-SE (5.26 g, 4.77 mmol, 0.4 equiv) was dissolved in anhydrous THF (30 mL) and slowly added dropwise to the above cooled lithium phosphide solution. After the addition was complete, the reaction mixture was stirred at −78 °C for another 1.5 h, after which the cooling bath was removed and the mixture was allowed to warm naturally to room temperature and stirred overnight [30].

##### Workup and Purification of L_TRPT22_

After completion of the above reaction, a mixture of glacial acetic acid (1 mL) and deionized water (15 mL) was added to the reaction system, and the mixture was stirred for 5 min. After standing for 3 min, the reaction mixture was transferred to a separatory funnel under a strict Ar atmosphere, and the upper organic phase was separated and collected. The retained aqueous phase was extracted with THF (20 mL × 3). The combined organic phases were concentrated under reduced pressure to remove THF, and toluene (40 mL) was then added to the residue. The resulting toluene mixture was cooled in an ice bath and then transferred to a separatory funnel under N_2_ protection, followed by the addition of deoxygenated deionized water (15 mL). The biphasic system was allowed to stand overnight at low temperature. After phase separation, the aqueous phase was collected, and the retained toluene phase was back-extracted with deionized water (20 mL × 3). Finally, the combined aqueous phases were concentrated under reduced pressure with gentle heating to remove water, affording a white waxy mixture (4.14 g). Based on the NMR spectrum (Figure S5) analysis, the mass content of the target product is 10.86%, meaning that the actual mass of L_TRPT22_ is 0.4496 g, corresponding to a yield of 2.6%.

#### 3.2.2. Preparation of the L_TRPT22_-Pd(0) Nanocatalyst

In a PTFE-lined stainless-steel autoclave, an aqueous solution of Na_2_PdCl_4_ (10.5 mg, 0.0356 mmol, 0.5 mL), deionized water (3.5 mL), L_TRPT22_ (102.0 mg, 0.0712 mmol), and toluene (2.0 mL) were added in sequence. After the autoclave was sealed, it was purged five times with hydrogen (1 MPa), and then charged with hydrogen to a pressure of 4 MPa. The reactor was placed in an oil bath preheated to 80 °C and stirred at 350 rpm for 3 h. After the reaction was completed, the autoclave was cooled in an ice bath for 2 h and then slowly depressurized. Upon opening the autoclave, the aqueous phase was observed to be dark brown, while the organic phase remained colorless and transparent, indicating the successful formation of the L_TRPT22_-Pd(0) nanocatalyst. The resulting biphasic system was directly used in the subsequent catalytic reactions [31]. Based on the ^1^H NMR spectrum (Figure S5) analysis, the 102.0 mg of L_TRPT22_ added was a mixture containing PEG-SE, and the actual mass of L_TRPT22_ was 11.08 mg (0.0077 mmol).

#### 3.2.3. Application of the L_TRPT22_-Pd(0) Nanocatalyst in the Catalytic Mizoroki–Heck Coupling Reaction and Solvent Screening

Under an Ar atmosphere, the above-prepared biphasic dispersion of the L_TRPT22_-Pd(0) nanocatalyst in toluene (2 mL)/water (4 mL) containing 0.0356 mmol of Pd (4.0 mol% relative to iodobenzene), iodobenzene (0.1 mL, 0.89 mmol), butyl acrylate (0.104 mL, 0.726 mmol), CuI (8.0 mg, 0.042 mmol), and triethylamine (0.24 mL, 1.72 mmol) were added sequentially to a pressure-resistant reaction bottle. The bottle was sealed and heated in an oil bath at 85 °C with vigorous stirring for 2.5 h. After the reaction was completed, the mixture was cooled to room temperature and allowed to stand until phase separation occurred. The upper organic phase was collected with a dropper and concentrated under reduced pressure to give a crude mixture, which was purified by silica gel column chromatography to afford butyl cinnamate (0.1367 g, 92.2% yield).

When toluene (6 mL), H_2_O (6 mL), *n*-pentanol (6 mL), or *n*-pentanol/H_2_O (2 mL/4 mL) was used as the solvent, the experimental procedure was the same as described above.

#### 3.2.4. Catalyst Recycling Experiments

After each reaction was completed, the upper organic phase was removed with a dropper. Toluene (2 mL) was added to the retained aqueous phase for washing, and the toluene layer was then removed. Iodobenzene (0.1 mL, 0.89 mmol), butyl acrylate (0.104 mL, 0.73 mmol), CuI (8.0 mg, 0.042 mmol), and triethylamine (0.24 mL, 1.72 mmol) were then re-added sequentially to the reaction bottle for the next run. Each reaction was carried out under the same conditions, and the post-treatment procedure was repeated as described in Section 3.2.3. This recycling process was repeated continuously for four runs, and the catalytic activity and recyclability of the L_TRPT22_-Pd(0) nanocatalyst were evaluated on the basis of product yield and TOF values.

##### Structural Characterization of the Compounds

PEG-SE. ^1^H NMR (600 MHz, CDCl_3_/TMS) δ 4.40–4.20 (q, 2H, CH_2_OSO_2_), 3.75–3.50 (m, 86H, OCH_2_CH_2_O), 3.31 (s, 3H, OCH_3_), 3.17–3.08 (m, 2H, CH_2_CH_3_), 1.36 (s, 3H, CH_2_CH_3_). ^13^C NMR (150 MHz, CDCl_3_/TMS) δ 71.90, 70.57, 69.07, 68.92, 58.98, 45.00, 8.15. IR (ATR) 2861 (C–H), 1464, 1342 (CH_2_ bending), 1240 (S=O), 1110, 1103 cm^−1^ (C–O–C).

Intermediate 2. ^1^H NMR (600 MHz, DMSO-d_6_/TMS) δ 7.71 (d, 2H, ArH), 7.51–7.27 (m, 3H, ArH), 7.03–6.84 (m, 3H, ArH), 6.58–6.50 (m, 1H, ArH), 3.67 (s, 6H, OCH_3_), 3.75–3.59 (m, 2H, CH), 2.92–2.79 (m, 1H, CH), 1.21 (d, 12H, CH_3_), 0.97 (d, 6H, CH_3_).

L_TRPT22_. ^1^H NMR (600 MHz, D_2_O) δ 7.88–7.72 (m, 5H, ArH), 7.69–7.58 (m, 3H, ArH), 7.56–7.54 (m, 1H, ArH), 3.90–3.89 (m, 8H, ArOCH_3_ + PCH_2_CH_2_O), 3.76–3.72 (m, 84H, OCH_2_CH_2_O), 3.47–3.40 (m, 6H, CH_2_OCH_3_ + ArCH), 2.99–2.89 (m, 2H, PCH_2_), 1.42 (d, 18H, CH_3_CHCH_3_). IR (ATR) 2869 (C–H), 1635 (Ar C=C), 1348 (CH_3_ bending), 1248 (Ar–O–CH_3_), 1092 (P–C), 710 cm^−1^ (P–Ph). ^31^P NMR (243 MHz, CDCl_3_) δ -15.52.

Butyl cinnamate. ^1^H NMR (600 MHz, Methanol-d_4_) δ 7.67–7.53 (m, 3H, ArH), 7.41–7.29 (m, 3H, ArH + ArCH=CH), 6.50 (d, 1H, ArCH=CH), 4.19 (t, 2H, CH_2_), 1.73–1.55 (m, 2H, CH_2_CH_2_CH_2_), 1.44–1.28 (m, 2H, CH_2_CH_3_), 0.96 (t, 3H, CH_3_). ^13^C NMR (150 MHz, Methanol-d_4_) δ 167.17, 144.64, 134.37, 130.09, 128.65, 127.87, 117.73, 64.10, 30.58, 18.89, 12.86.

#### 3.2.5. TEM Observation of the L_TRPT22_-Pd(0) Nanocatalyst

A small aliquot of the aqueous Pd nanocatalyst dispersion was mixed with an equal volume of absolute ethanol and sonicated for 15 min. A small amount of the resulting dispersion liquid was then dropped onto an ultrathin carbon-coated copper grid and dried under an infrared lamp. After solvent evaporation, several drops of hot absolute ethanol were applied to the grid to wash away residual organic impurities, followed by drying again under the infrared lamp. This washing and drying procedure was repeated 3 times. The prepared sample was then subjected to TEM for morphological observation and imaging. Subsequently, approximately 20 Pd NPs were randomly selected from the obtained TEM images to measure their sizes using image analysis software, and the corresponding particle size distribution histogram was plotted.

## 4. Conclusions

In this study, a novel phosphine ligand, L_TRPT22_, was developed, and its thermoregulated phase-transfer property was successfully utilized to achieve both the catalytic application and recovery of the L_TRPT22_-Pd(0) nanocatalyst. The bulky electron-rich arylphosphine structure of the ligand is beneficial for enhancing the activity and stability of the catalyst, while the PEG chain imparts recyclability to the catalytic system. The L_TRPT22_-Pd(0) nanocatalyst exhibited good catalytic activity in the Mizoroki–Heck reaction under conventional conditions. Notably, the catalyst could be readily recovered by simple phase separation and still maintain relatively stable performance after four consecutive recycling runs. Through a ligand design strategy that simultaneously improves catalytic activity, stability, and recovery efficiency, this study provides a feasible approach for developing novel efficient and recyclable homogeneous nanopalladium catalysts.

## Figures and Tables

**Figure 1:**
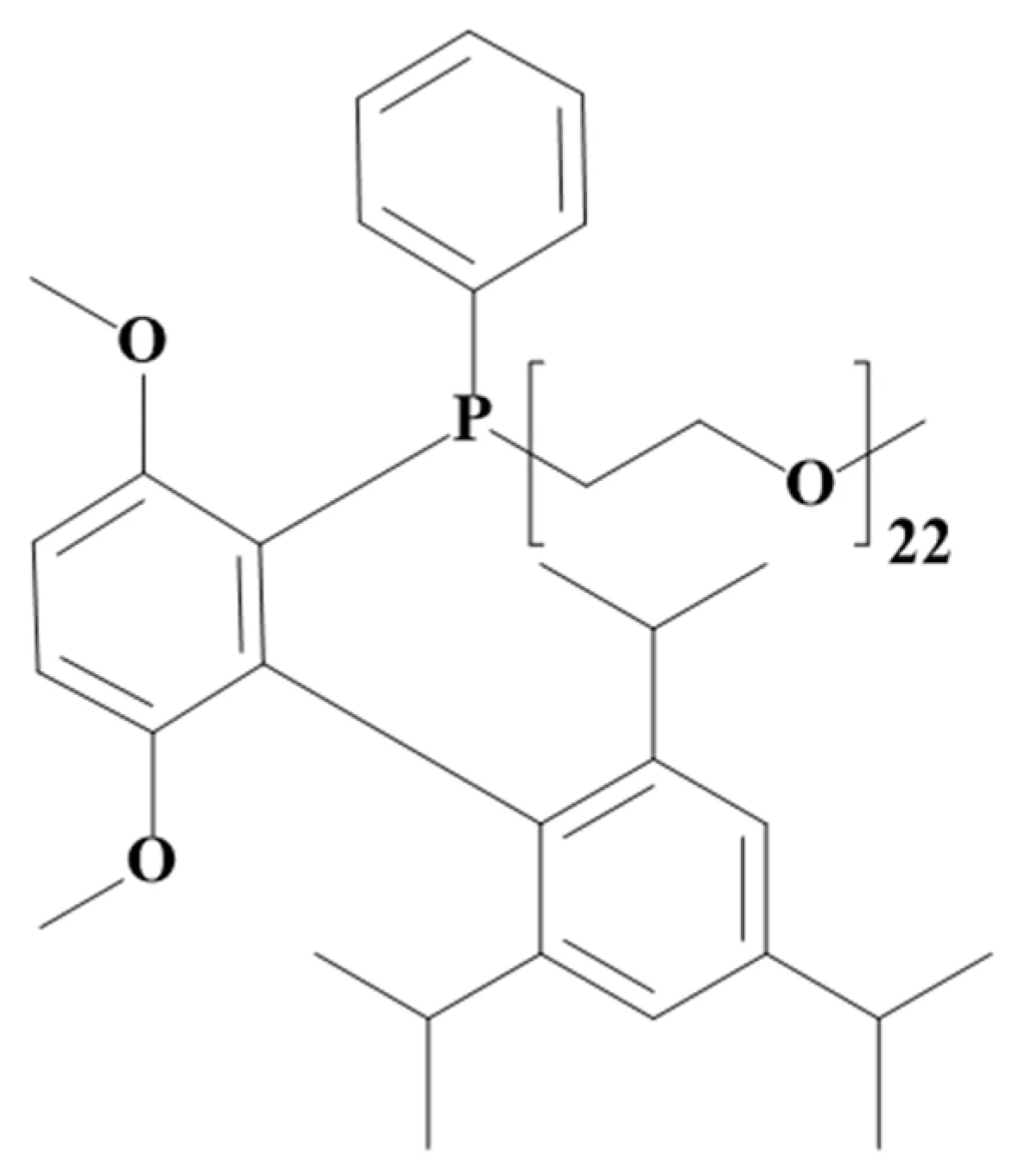
Molecular structure of L_TRPT22_.

**Figure 2:**
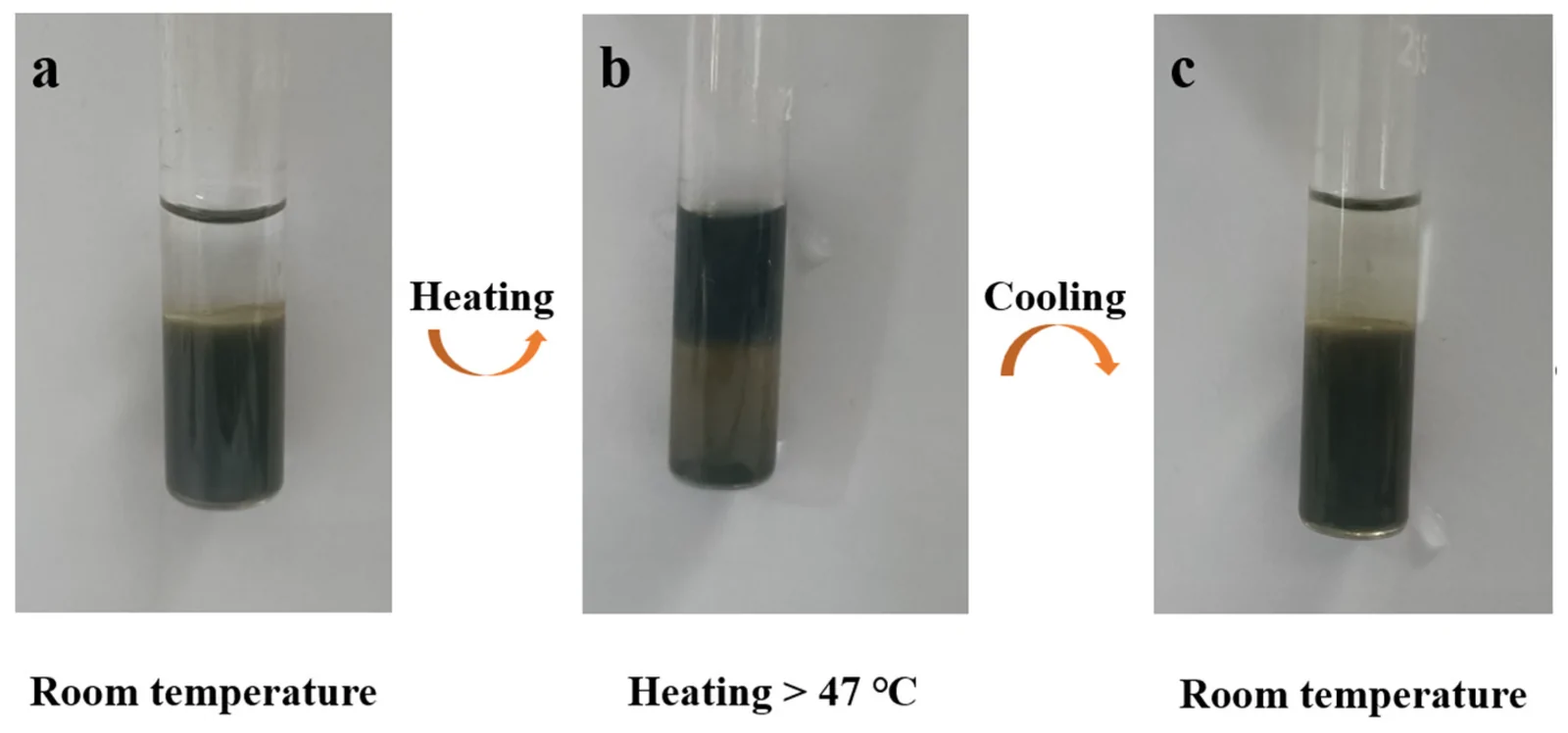
Thermoregulated phase-transfer process of the L_TRPT22_-Pd(0) nanocatalyst: (**a**) before heating, (**b**) after heating, (**c**) after cooling.

**Figure 3:**
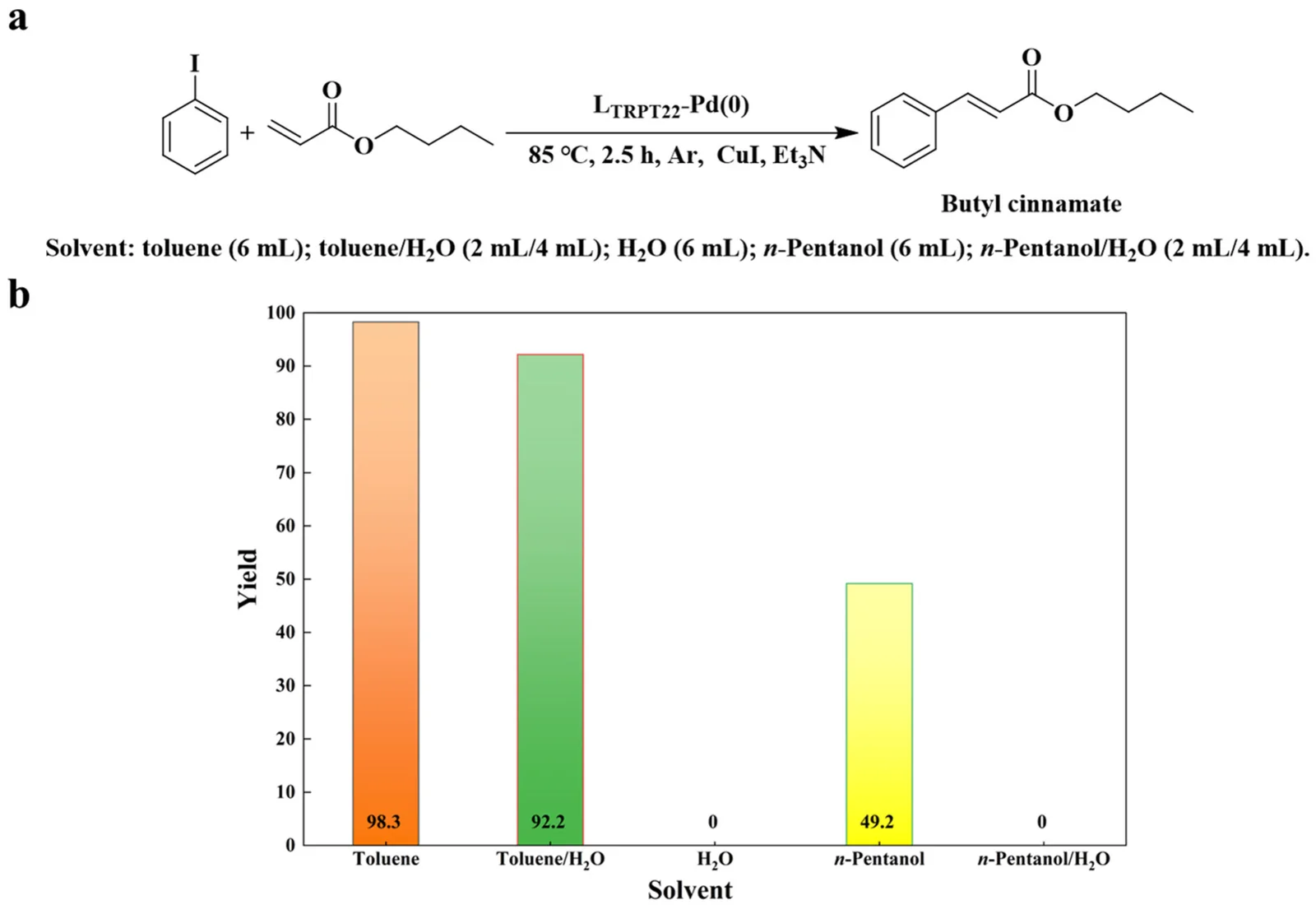
(**a**) Mizoroki–Heck coupling of iodobenzene with butyl acrylate. (**b**) Effect of different solvents on the reaction yield.

**Figure 4:**
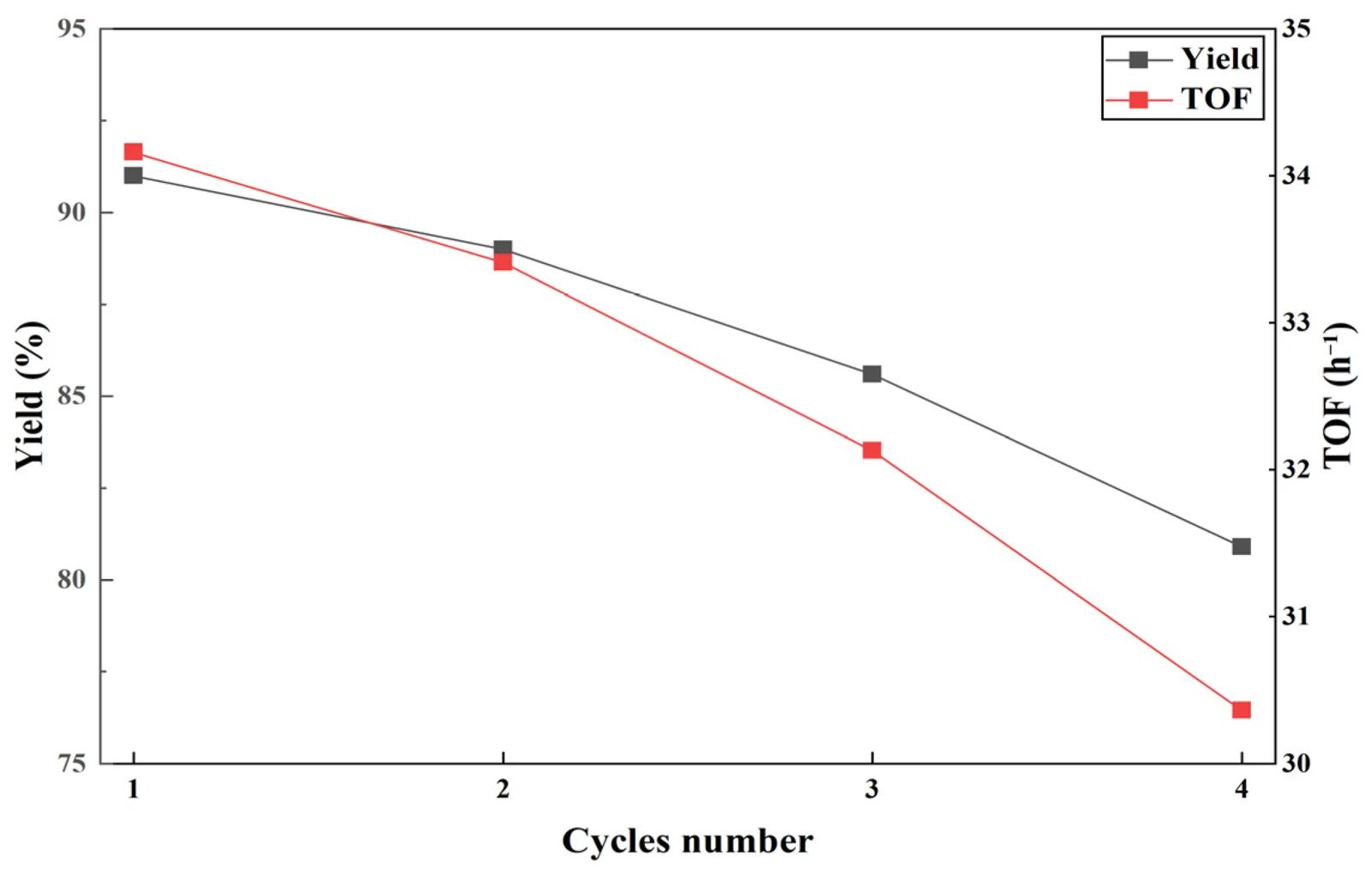
Effect of the number of recycling runs on the yield and TOF values. ($TOF=\frac{n_{product}}{n_{Pd}\times t}$).

**Figure 5:**
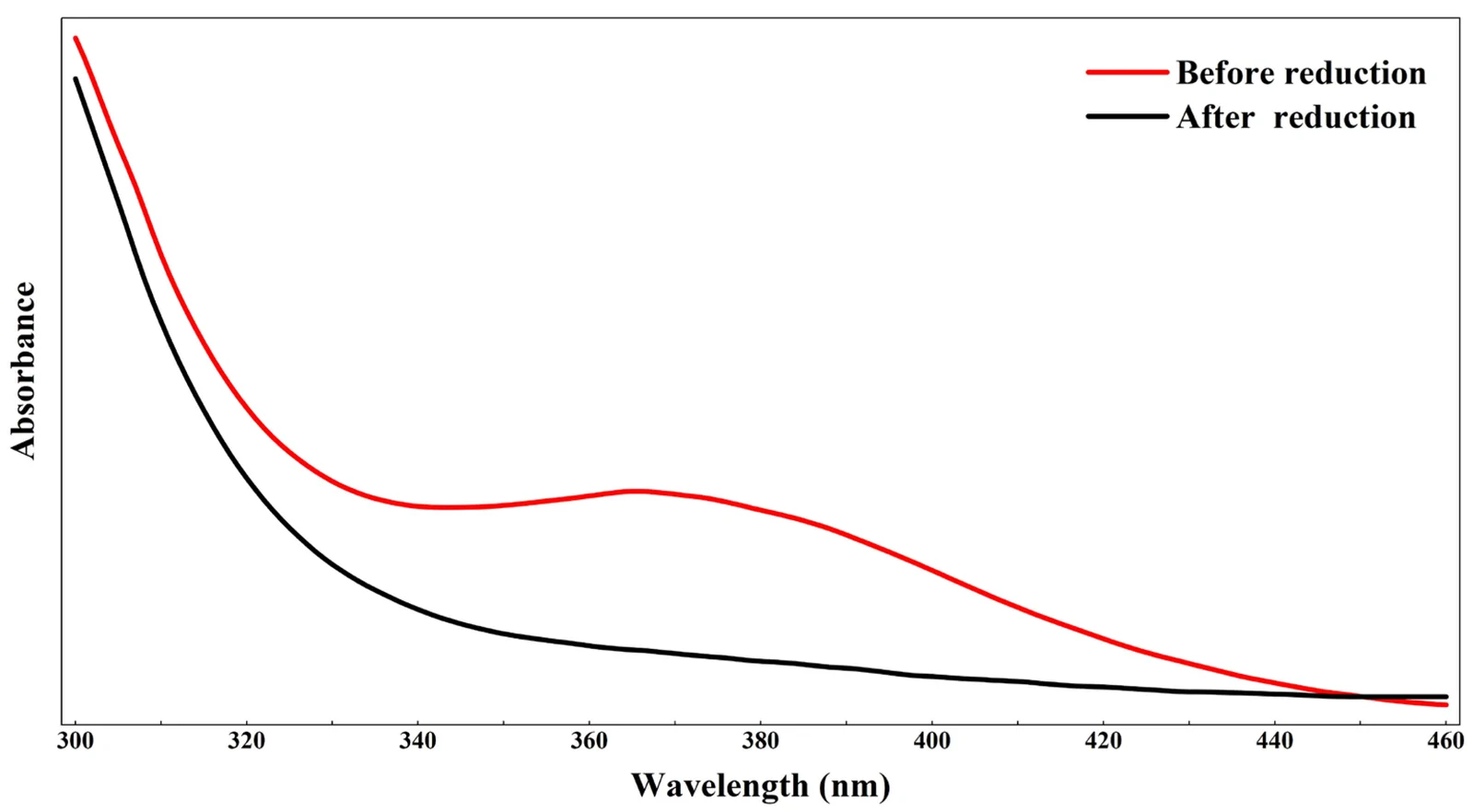
UV–Vis absorption spectra of Na_2_PdCl_4_ and L_TRPT22_ in H_2_O.

**Figure 6:**
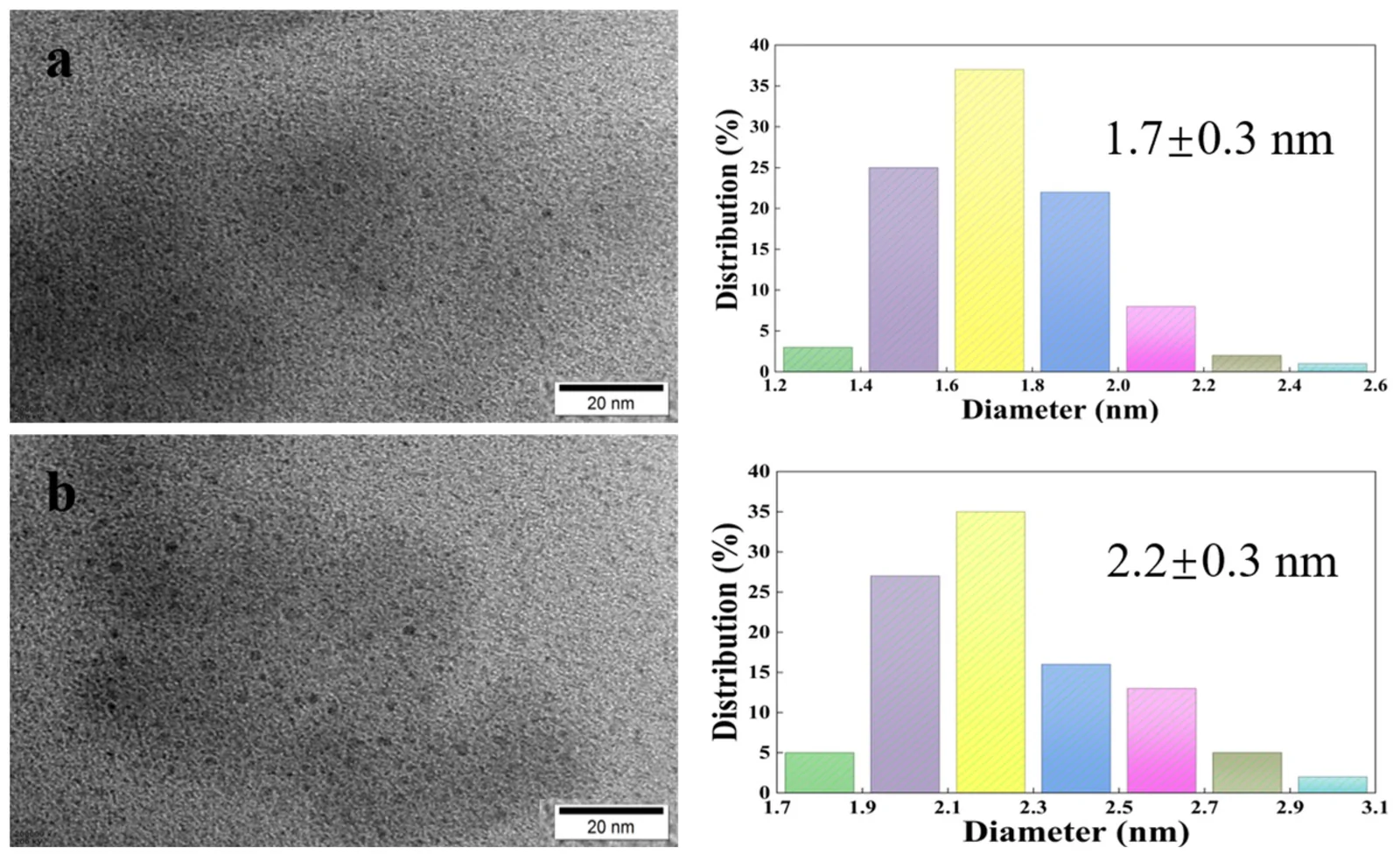
TEM image and particle size distribution of L_TRPT22_-Pd(0) nanocatalyst: (**a**) newly prepared; (**b**) after 4 cycles of use.

**Scheme 1:**
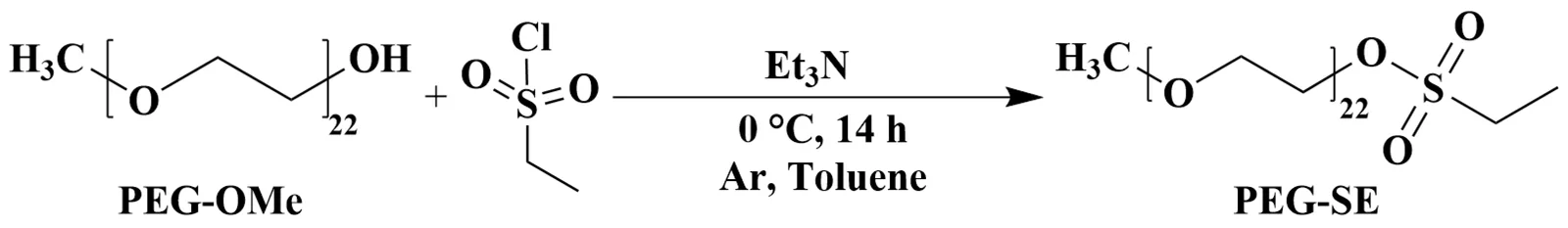
Synthetic route of PEG-SE.

**Scheme 2:**
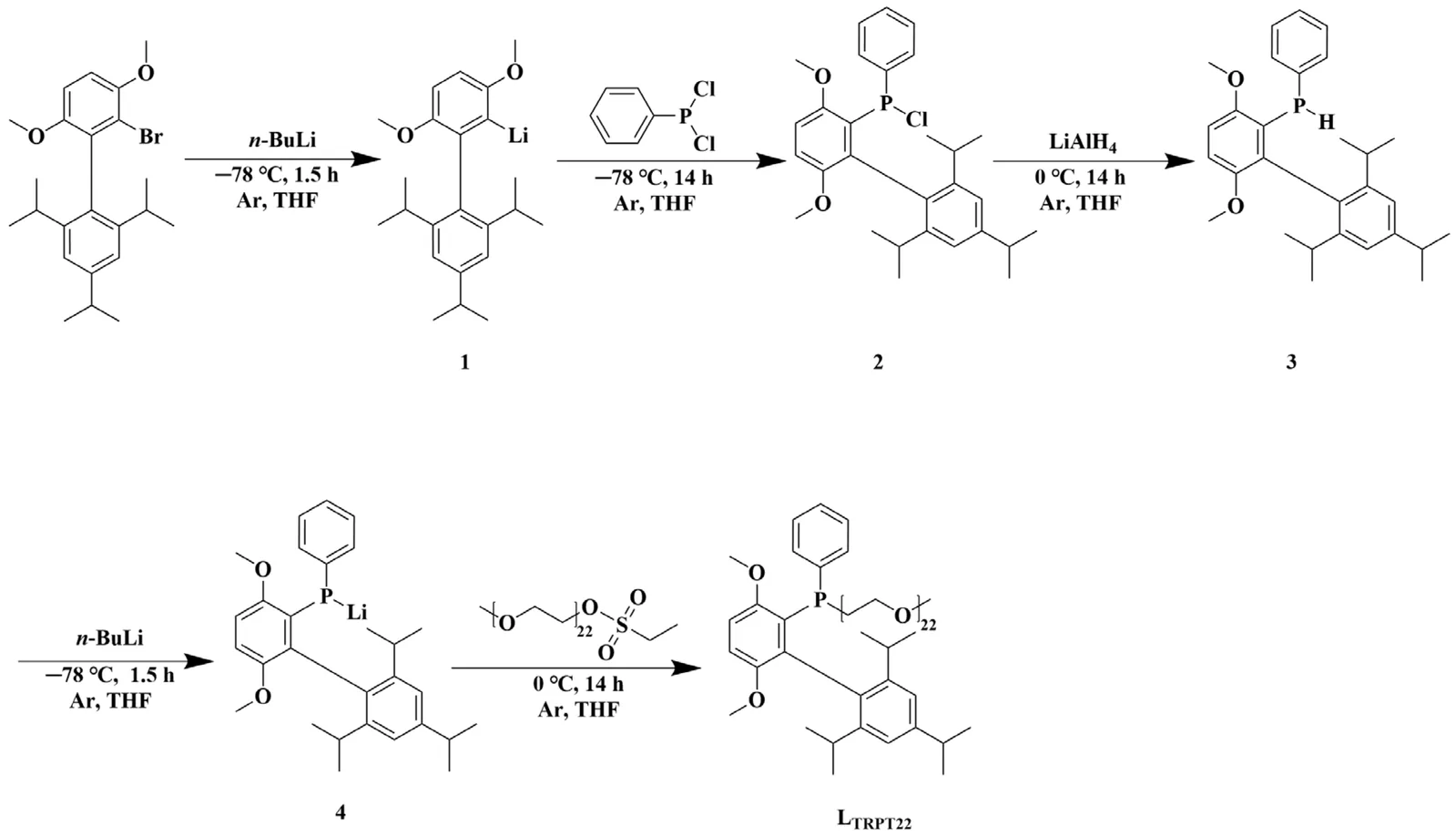
Synthetic route of L_TRPT22_.

## Data Availability

The data presented in this study are available in the article and Supplementary Materials.

## Supplementary Materials

The following supporting information can be downloaded at: https://www.mdpi.com/article/10.3390/molecules31193454/s1, Figure S1: ^1^H NMR spectrum of PEG-SE; Figure S2: ^13^C NMR spectrum of PEG-SE; Figure S3: FTIR spectrum of PEG-SE; Figure S4: ^1^H NMR spectrum of intermediate 2; Figure S5: ^1^H NMR spectrum of L_TRPT22_; Figure S6: ^31^P NMR spectrum of L_TRPT22_; Figure S7: FTIR spectrum of L_TRPT22_; Figure S8: ^1^H NMR spectrum of butyl cinnamate; Figure S9: ^13^C NMR spectrum of butyl cinnamate; Table S1: Comparison with the reported TRPT catalysts and some other related catalysts.
