# Supplementary material for: Synthesis and Catalytic Application of a Novel Recyclable Thermoregulated Phase-Transfer Phosphine Ligand
Source: Molecules. 2026 Sep 28;31(19):3454. doi: 10.3390/molecules31193454 (PMC13666364; doi:10.3390/molecules31193454)
Supplement: Supplementary file 1 [file molecules-31-03454-s001.zip › molecules-4547529-supplementary.pdf]

Supplementary Material

for

Synthesis and Catalytic Application of a Novel Recyclable Thermoregulated Phase-Transfer Phosphine Ligand

Yang Liu [1,2,*], Tianyuan Shao [1], Zhenghui Ma [1], Rui Wu [1,2] and Guanghui Tian [1,2]

[1] School of Chemistry and Environment Science, Shaanxi University of Technology, Hanzhong 723001, China

[2] Shaanxi Key Laboratory of Catalysis, Shaanxi University of Technology, Hanzhong 723001, China

* Correspondence: liuyang@snut.edu.cn (Y.L.)

CONTENTS

Figure S1. ^{1}H NMR spectrum of **PEG-SE** 1

Figure S2. ^{13}C NMR spectrum of **PEG-SE** 1

Figure S3. FTIR spectrum of **PEG-SE** 2

Figure S4. ^{1}H NMR spectrum of **intermediate 2** 2

Figure S5. ^{1}H NMR spectrum of $\mathbf{L_{TRPT22}}$ 3

Figure S6. ^{31}P NMR spectrum of $\mathbf{L_{TRPT22}}$ 4

Figure S7. FTIR spectrum of $\mathbf{L_{TRPT22}}$ 5

Figure S8. ^{1}H NMR spectrum of **butyl cinnamate** 5

Figure S9. ^{13}C NMR spectrum of **butyl cinnamate** 6

Table S1. Comparison with the reported TRPT catalysts and some other related catalysts 7

Supplementary Figures

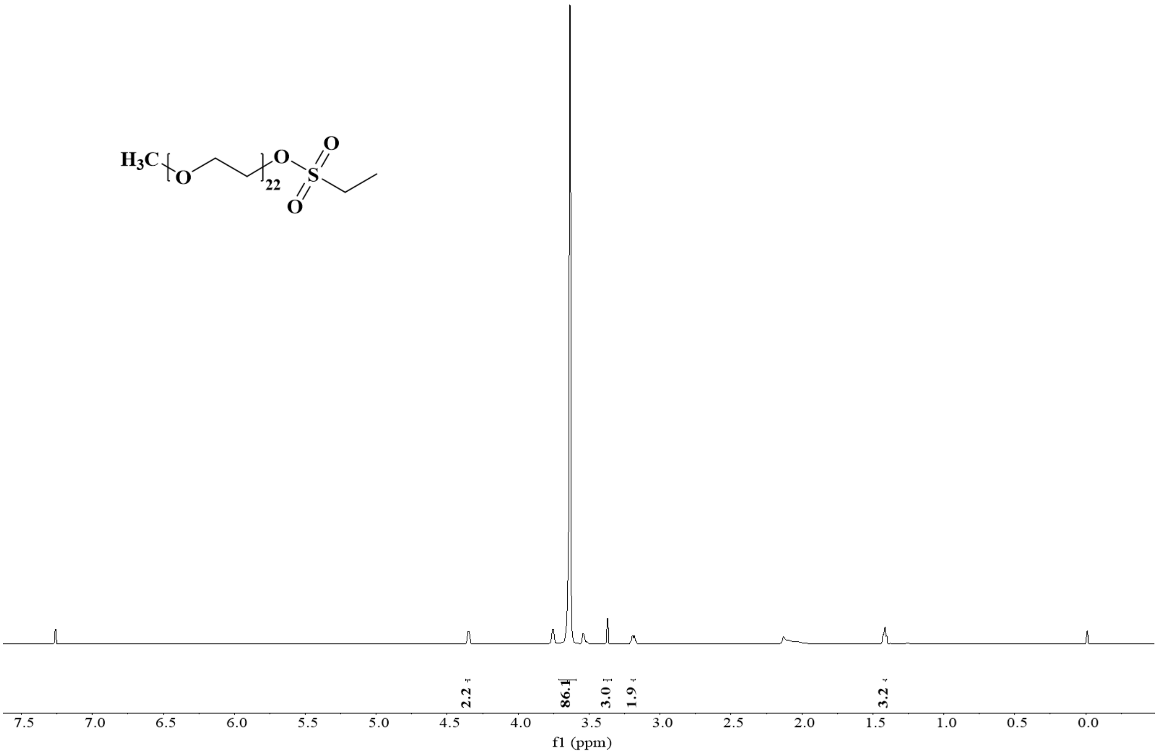

Figure S1. ^{1}H NMR spectrum of **PEG-SE**.

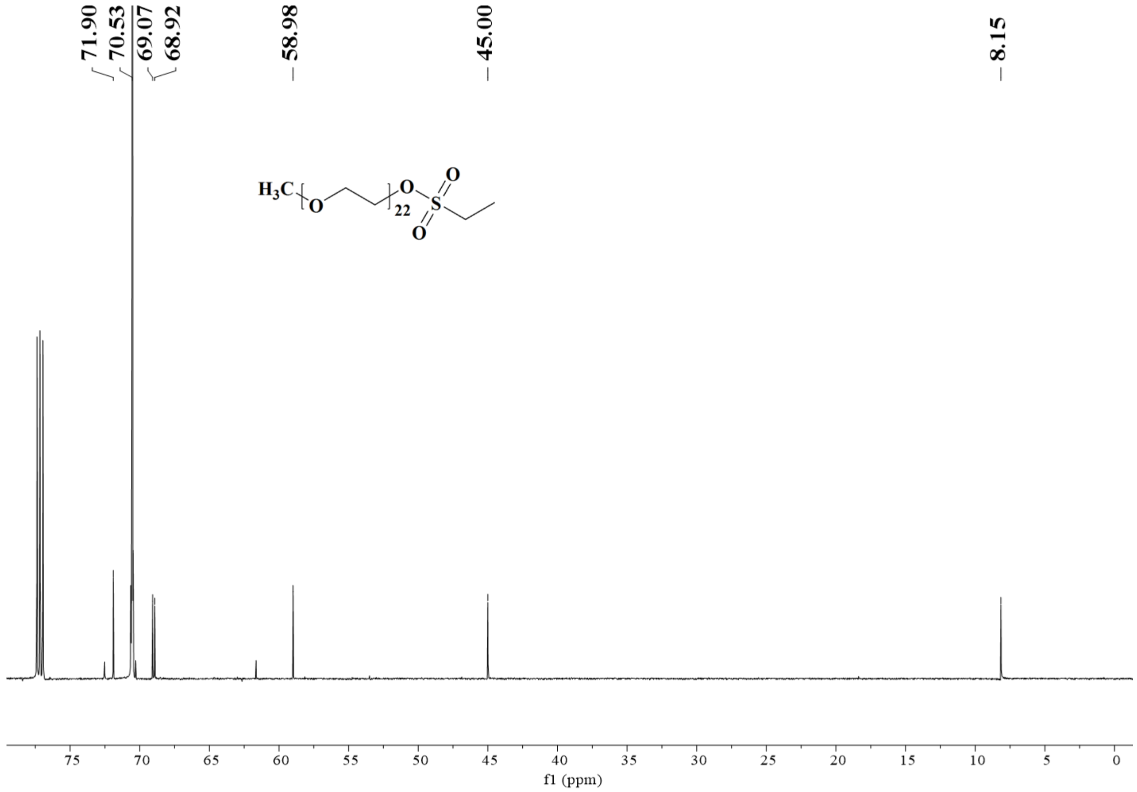

Figure S2. ^{13}C NMR spectrum of **PEG-SE**.

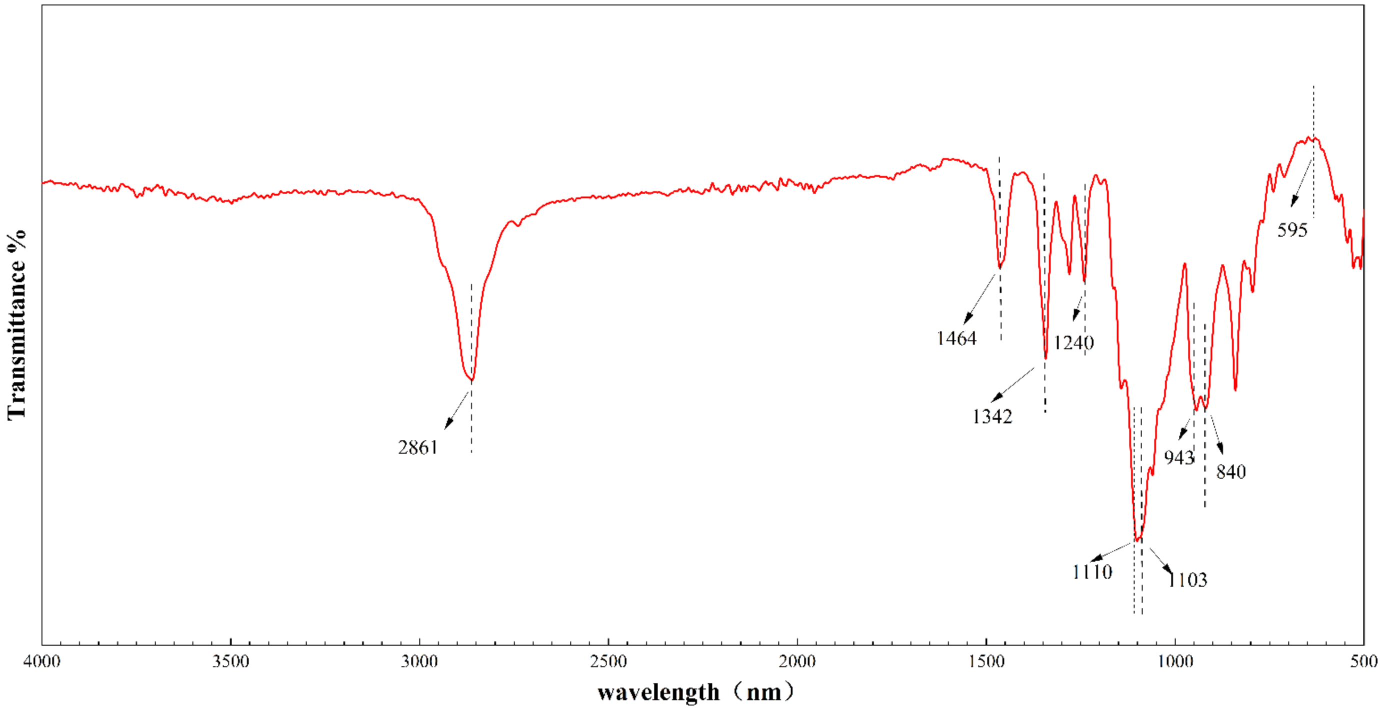

Figure S3. FTIR spectrum of **PEG-SE**.

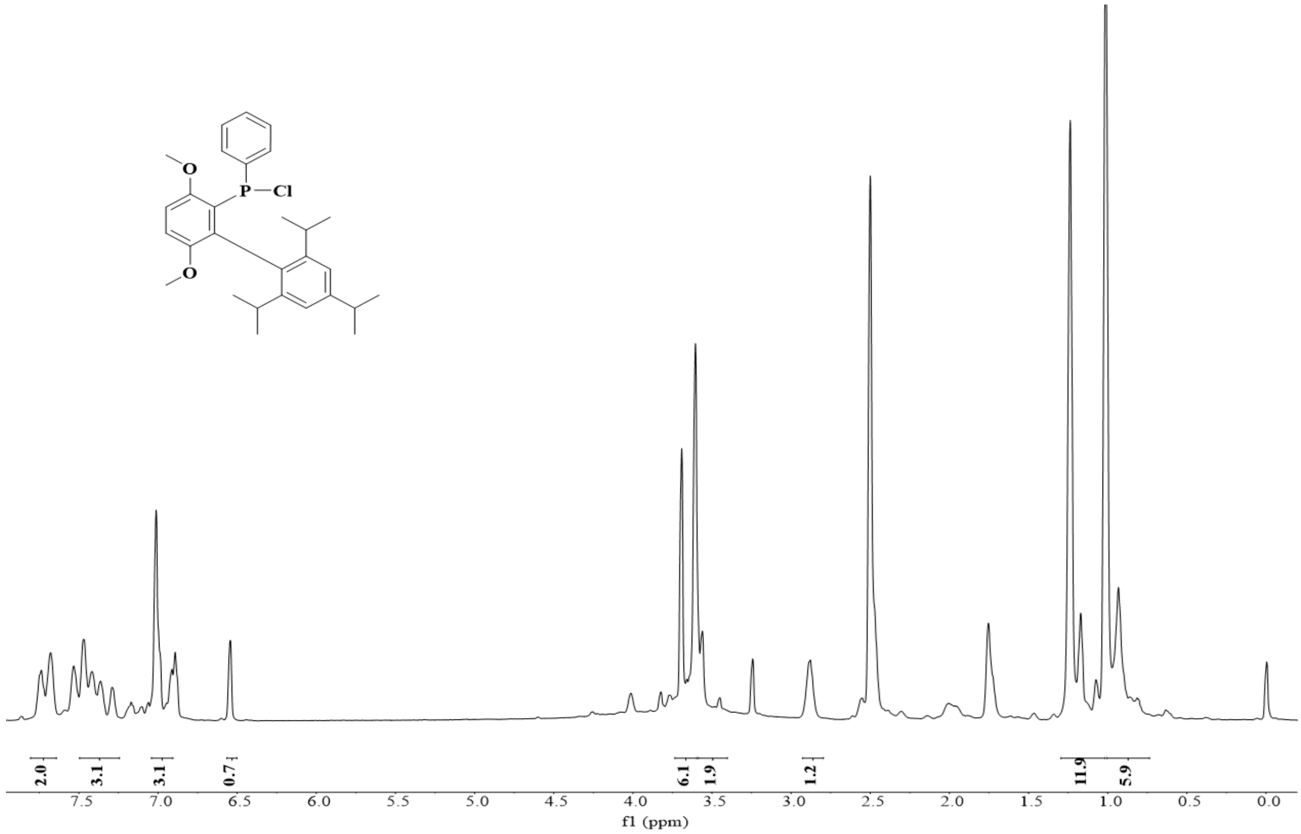

Figure S4. ^{1}H NMR spectrum of intermediate 2.

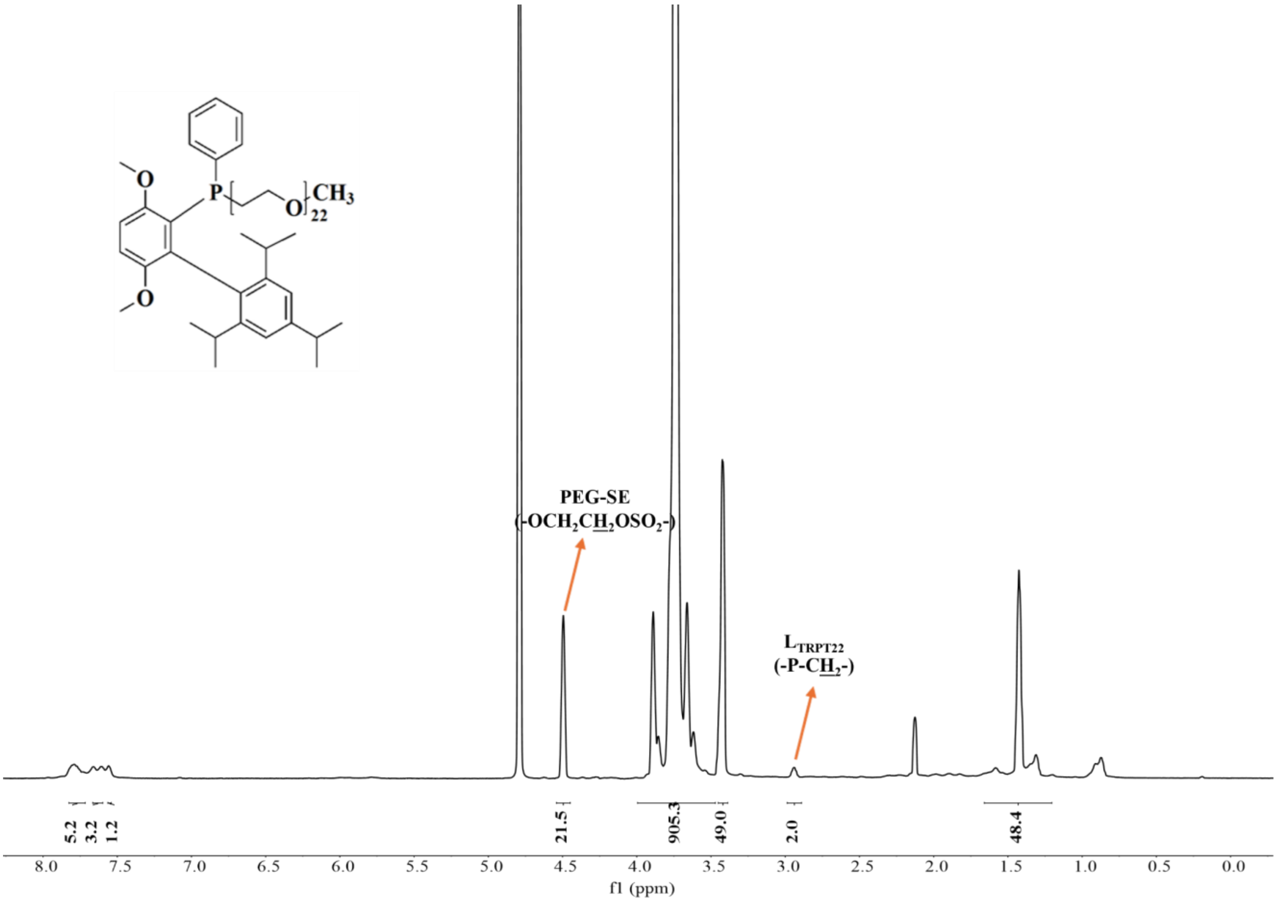

Figure S5. ^{1}H NMR spectrum of **L$_{TRPT22}$.**

Note on Figure S5 integration and product content calculation: Due to the inherent difficulty in separating the amphiphilic L$_{TRPT22}$ ligand from unreacted PEG-SE by standard chromatography, the obtained white waxy material (4.14 g) is a mixture. To estimate the actual content of L$_{TRPT22}$, the ^{1}H NMR integration was carefully calibrated. The isolated signal of the P-CH_2 protons of L$_{TRPT22}$ (set to 2 H) was used as the reference. By comparing it with the characteristic signal of the -CH_2OSO_2- protons from the residual PEG-SE (integrated to 21.5 H), the molar ratio of L$_{TRPT22}$ to PEG-SE was determined to be 1:10.75. Based on their respective molecular weights (Mw of L$_{TRPT22}$ = 1431.78; Mw of PEG-SE = 1093.32), the mass content of the target compound L$_{TRPT22}$ in the mixture is calculated to be 10.86%, and the corresponding mass was 0.4496 g. This can afford an estimated isolated yield of 2.6% based on the starting material 2-bromo-2′,4′,6′-triisopropyl-3,6-dimethoxy-1,1′-biphenyl. The signals at 3.9-3.8, 3.47–3.40, and 1.4 ppm all involve the overlapping of non-characteristic signal peaks with similar chemical shifts from both L$_{TRPT22}$ and PEG-SE. The integral values of the protons in these regions generally match the calculated results based on the aforementioned 1:10.75 molar ratio of L$_{TRPT22}$ to PEG-SE. Since both the starting material and the product are polymers, there will be some deviations between the actual integral values and the theoretical calculated values.

The calculation verification is as follows. (1) The region at 3.9–3.8 ppm contains the aromatic methoxy protons of L$_{TRPT22}$ (6 H, $ArOCH_3$), the polyether chain methylene protons (86 H), and the polyether chain methylene protons of PEG-SE (86 × 10.75 H). Theoretically, there should be 6 + 86 + 86 × 10.75 = 1016.5 H in this region, while the

actual integral is 905.3 H. (2) The region at 3.47–3.40 ppm contains the polyether chain methoxy protons (3 H, $CH_2OC\underline{H}_3$) and aromatic methine protons (3 H, ArCH) of LTRPT22, as well as the polyether chain methoxy protons of PEG-SE (3 × 10.75 H, $CH_2OC\underline{H}_3$). Theoretically, there should be 3 + 3 + 3 × 10.75 = 38.25 H in this region, while the actual integral is 49 H. (3) The region at 1.4 ppm contains the isopropyl protons of the product (18 H, $C\underline{H}_3CHC\underline{H}_3$) and the terminal methyl protons of PEG-SE (3 × 10.75 H). Theoretically, there should be 18 + 3 × 10.75 = 50.25 H in this region, while the actual integral is 48.4 H. (4) The signal peak at 2.1-2.2 ppm is generated by impurities contained in the starting material PEG-SE (Figure S1). No matter how strictly control the reaction conditions and purify the product, the synthesized starting material PEG-SE will always produce a minor impurity peak at 2.1-2.2 ppm, and this impurity peak becomes slightly more obvious during the subsequent synthesis of LTRPT22.

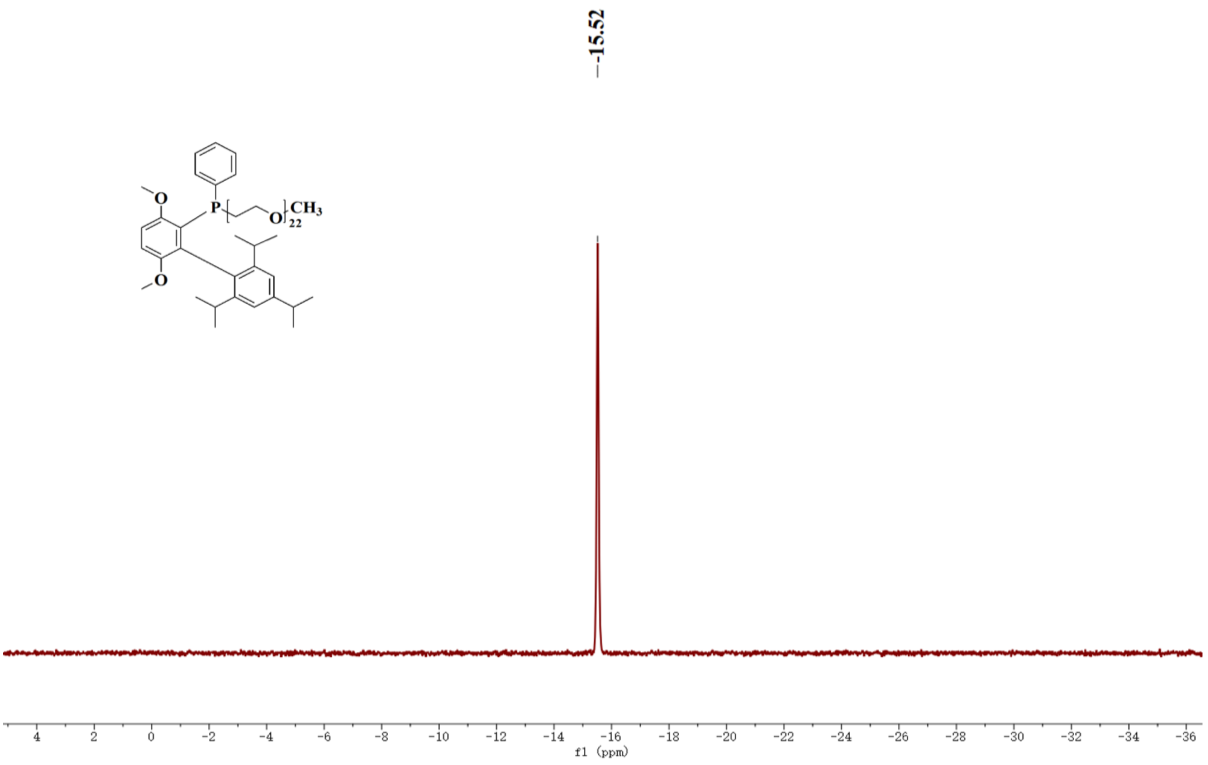

Figure S6. ^{31}P NMR spectrum of **LTRPT22.**

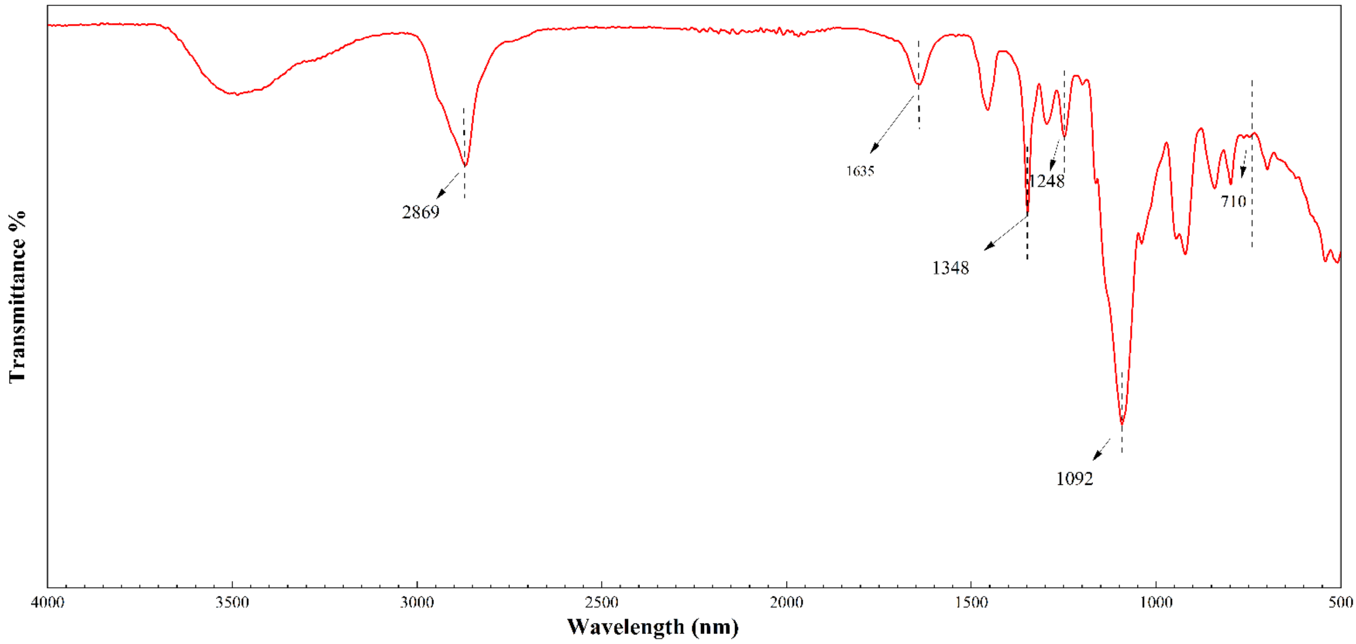

Figure S7. FTIR spectrum of **LTRPT22**.

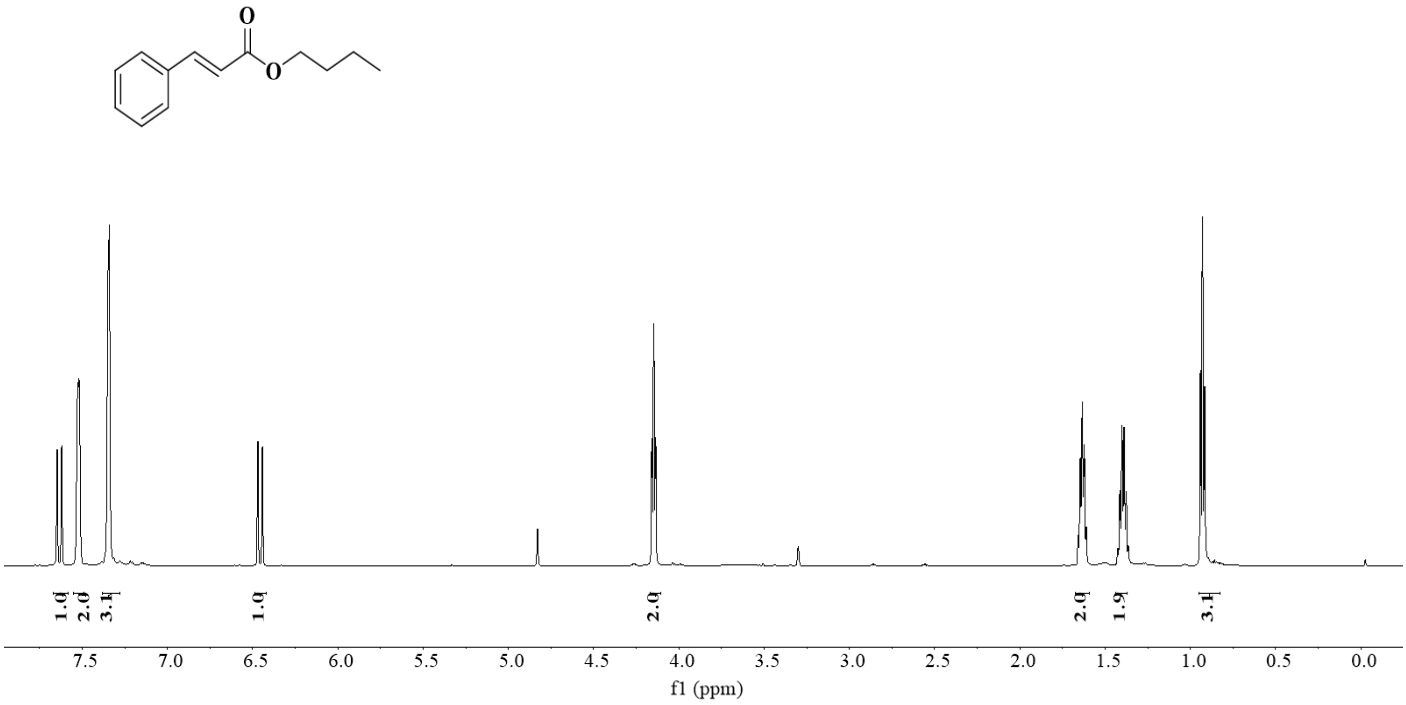

Figure S8. ^{1}H NMR spectrum of **butyl cinnamate**.

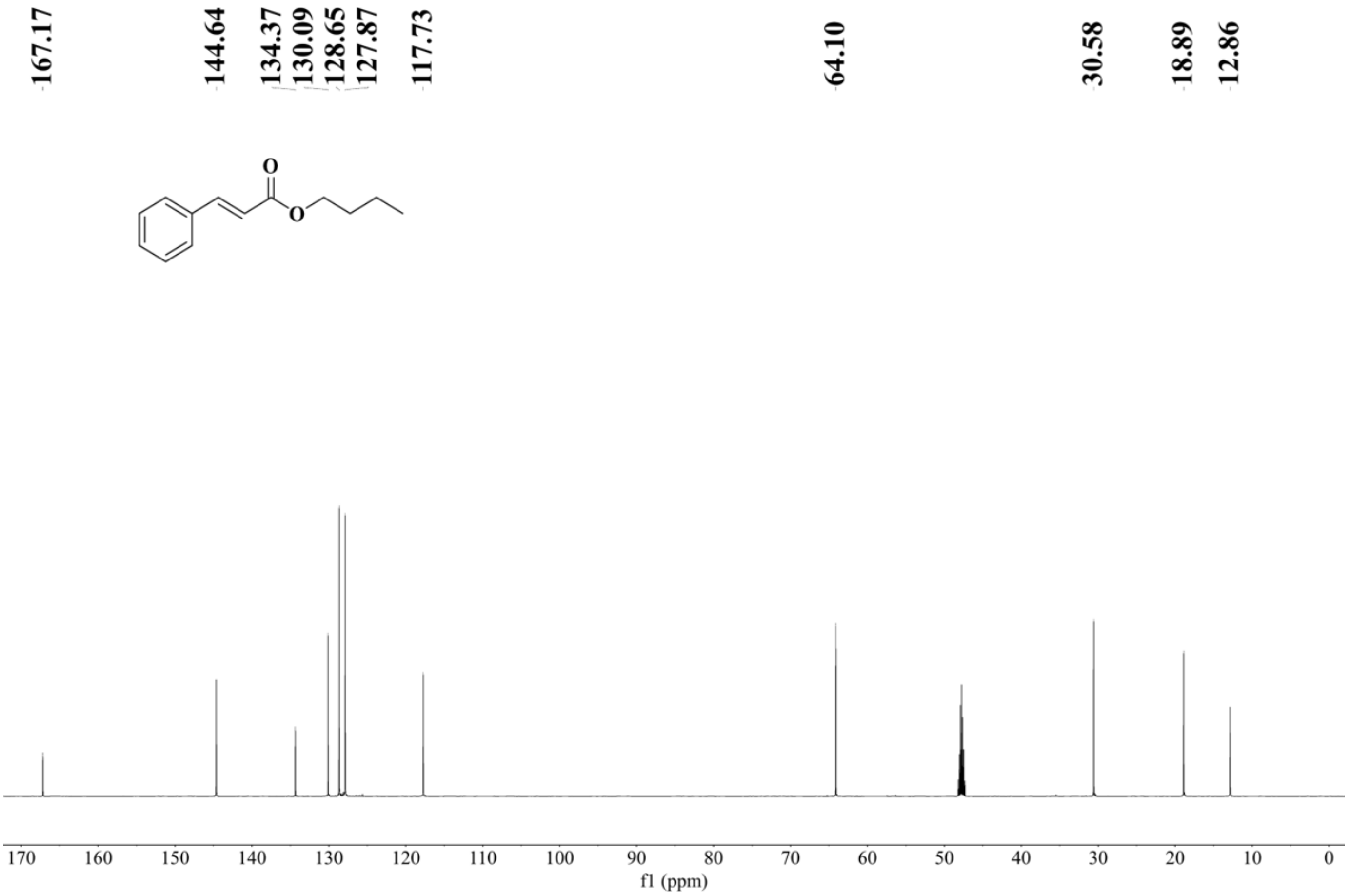

Figure S9. ^{13}C NMR spectrum of **butyl cinnamate**.

Table S1. Comparison with the reported TRPT catalysts and some other related catalysts.

No.	Ligand or catalyst structure (type)	Metal center	Reaction type	Highest TOF (h^{-1})	Recycling methods / frequency	Reference
Present work	L_{TRPT22} (TRPT)	Pd(0)	Heck coupling	34.2	Phase separation via cooling / 4 times	This contribution
1	$Ph_2P(CH_2CH_2O)_{22}CH_3$ (TRPT)	Ru(0)	Atmospheric hydrogenation of α,β-unsaturated ketones	49	Phase separation via cooling / 6 times	*Chin. J. Appl. Chem.* **2024**, *41*, 1350–1356.
2	$Ph_2P(CH_2CH_2O)_{22}CH_3$ (TRPT)	Pd(0)	Selective hydrogenation of 1,5-cyclooctadiene	1411	Phase separation via cooling / 5 times	*Catal. Lett.* **2020**, *150*, 2703–2708.
3	$Ph_2P(CH_2CH_2O)_{22}CH_3$ (TRPT)	Pd(0)	Atmospheric hydrogenation of α,β-unsaturated ketones	870	Phase separation via cooling / 4 times	*Catal. Commun.* **2019**, *125*, 10–14.
4	Pd@MicroCS (Chitosan microspheres)	Pd NPs	Heck coupling	NM	Filtration / 14 times	*New J. Chem.* **2025**, *49*, 17370–17377.
5	Pd-10/N-C (Nitrogen-doped carbon)	Pd NPs and clusters	Heck coupling	792.5	Centrifugation / NM	*J. Mol. Struct.* **2024**, *1316*, 139081.
6	$Pd_{0.1}Cu_{0.9}Co_2O_4$ (Nano-flakes)	Pd alloy	Heck & Suzuki coupling	NM	Centrifugation / 10 times	*Front. Chem.* **2024**, *12*, 1496234.
7	UiO-66-PPh_2–Pd (MOF-supported)	Pd(0)	Heck & Suzuki coupling	NM	Centrifugation / 4 times	*ChemistryOpen* **2024**, *13*, e202300249.
8	Pd(0)-SMTU-boehmite	Pd(0)	Heck & Suzuki coupling	NM	Filtration / 7 times	*Nanoscale Adv.* **2023**, *5*, 4925–4933.
9	Pd–ZnO–Scb (Schiff base-modified ZnO)	Pd NPs	Heck coupling	NM	Centrifugation / 7 times	*Sci. Rep.* **2023**, *13*, 12008.
10	Pd@ASP–EDTA–CS (Chitosan nanocomposite)	Pd NPs	Heck coupling	NM	Filtration / 5 times	*Nanoscale Adv.* **2023**, 5, 2621–2638.

No.	Ligand or catalyst structure (type)	Metal center	Reaction type	Highest TOF (h^{-1})	Recycling methods / frequency	Reference
Present work	L_{TRPT22} (TRPT)	Pd(0)	Heck coupling	34.2	Phase separation via cooling / 4 times	This contribution
11	Pd/Cu on charcoal (Heterogeneous multi-task catalyst)	Pd/Cu NPs	Sequential Sonogashira–Click & Click–Heck reactions	NM	Filtration / 4 times	*J. Organomet. Chem.* **2014**, *755*, 78–85.
12	KCC-1-NH_2/Pd (Fibrous nano-silica-supported Pd)	Pd complex	Heck, Suzuki, Sonogashira	NM	Filtration / 7 times	*ChemSusChem* **2012**, *5*, 85–89.
13	MOF-Cu(btc)-[Pd] (Metal-organic framework supported catalyst)	Pd/Cu bimetallic	Heteroannulation of acetylenic compounds	NM	Filtration / 10 times	*Adv. Synth. Catal.* **2012**, *354*, 1347–1355.

NM: Not Mentioned. Although the TOF values or recycling times of some solid-supported heterogeneous catalysts might be higher, the present L_{TRPT22}-Pd(0) catalyst operates homogeneously at elevated temperatures (ensuring excellent mass transfer) and separates spontaneously into the aqueous phase upon cooling. This TRPT strategy completely avoids the time-consuming solid-liquid separation steps (such as centrifugation, filtration, or membrane washing) and potential mechanical loss of the catalyst, presenting a highly green and operationally simple approach for palladium recycling.
